# Capturing artificial intelligence applications’ value proposition in healthcare – a qualitative research study

**DOI:** 10.1186/s12913-024-10894-4

**Published:** 2024-04-03

**Authors:** Jasmin Hennrich, Eva Ritz, Peter Hofmann, Nils Urbach

**Affiliations:** 1 FIM Research Institute for Information Management, University of Bayreuth, Branch Business and Information Systems Engineering of the Fraunhofer FIT, Wittelsbacherring 10, 95444 Bayreuth, Germany; 2 University St. Gallen, Dufourstrasse 50, 9000 St. Gallen, Switzerland; 3 Faculty Business and Law, Frankfurt University of Applied Sciences, Nibelungenplatz 1, 60318 Frankfurt Am Main, Germany; 4 appliedAI Initiative GmbH, August-Everding-Straße 25, 81671 Munich, Germany

**Keywords:** Artificial intelligence, Value propositions, Business objectives, Healthcare

## Abstract

Artificial intelligence (AI) applications pave the way for innovations in the healthcare (HC) industry. However, their adoption in HC organizations is still nascent as organizations often face a fragmented and incomplete picture of how they can capture the value of AI applications on a managerial level. To overcome adoption hurdles, HC organizations would benefit from understanding how they can capture AI applications’ potential.

We conduct a comprehensive systematic literature review and 11 semi-structured expert interviews to identify, systematize, and describe 15 business objectives that translate into six value propositions of AI applications in HC.

Our results demonstrate that AI applications can have several business objectives converging into risk-reduced patient care, advanced patient care, self-management, process acceleration, resource optimization, and knowledge discovery.

We contribute to the literature by extending research on value creation mechanisms of AI to the HC context and guiding HC organizations in evaluating their AI applications or those of the competition on a managerial level, to assess AI investment decisions, and to align their AI application portfolio towards an overarching strategy.

**Supplementary Information:**

The online version contains supplementary material available at 10.1186/s12913-024-10894-4.

## Background

### Overview

Applications based on artificial intelligence (AI) have the potential to transform the healthcare (HC) industry [1]. AI applications can be characterized as applications or agents with capabilities that typically demand intelligence [2, 3]. In our context, we understand AI as a collection of technological solutions from the field of applied computer science, in which algorithms are trained on medical and HC data to perform tasks that are normally associated with human intelligence (i.e., medical decision-making) [4]. AI is not a single type of technology, instead, it encompasses a diverse array of technologies spread across various application areas in HC, such as diagnostics (e.g., [5], biomedical research (e.g., [6], clinical administration (e.g., [7], therapy (e.g., [8], and intelligent robotics (e.g., [9]. These areas are expected to benefit from AI applications’ capabilities, such as accuracy, objectivity, rapidity, data processing, and automation [10, 11]. Accordingly, AI applications are said to have the potential to drive business value and enhance HC [12], paving the way for transformative innovations in the HC industry [13]. There are already many promising AI use cases in HC that are expected to improve patient care and create value for HC organizations. For instance, AI applications can advance the quality of patient care by supporting radiologists with more accurate and rapid diagnosis, compensating for humans’ limitations (e.g., data processing speeds) and weaknesses (e.g., inattention, distraction, and fatigue) [10, 14]. Klicken oder tippen Sie hier, um Text einzugeben.While the use of AI applications in HC has the overarching goal of creating significant value for patients through improved care, they also come with the potential for business value creation and the opportunity for HC organizations to gain a competitive edge (e.g., [15, 16]).

Despite the promised advantages, AI applications’ implementation is slow, and the full realization of their potential within the HC industry is yet to be achieved [11, 17]. With just a handful of practical examples of AI applications in the HC industry [13, 18], the adoption of AI applications is still in its infancy. The AI in Healthcare Survey Report stated that in 2021, only 9% of respondents worldwide have reached a sophisticated adoption of AI Models, while 32% of respondents are still in the early stages of adopting AI models. According to the survey, the majority of HC organizations (60%) are not actively considering AI as a solution, or they are currently evaluating AI use cases and experimenting with the implementation [19]. Nevertheless, HC startups are increasingly entering the market [20], pressuring incumbent HC organizations to evaluate and adopt AI applications. Existing studies already investigate AI technologies in various use cases in HC and provide insights on how to design AI-based services [21], explain in detail the technical functions and capabilities of AI technologies [10, 11], or take on a practical perspective with a focus on concrete examples of AI applications [14]. However, to foster the adoption of AI applications, HC organizations should understand how they can unfold AI applications’ capabilities into business value to ensure effective investments. Previous studies on the intersection of information systems and value creation have expressed interest into how organizations can actually gain value through the use of technology and thus, enhance their adoption [22, 23]. However, to the best of our knowledge, a comprehensive investigation of the value creation of AI applications in the context of HC from a managerial level is currently missing. Thus, our study aims to investigate AI applications’ value creation and capture mechanisms in the specific HC context by answering the following question: How can HC organizations create and capture AI applications’ value?

We conduct a systematic literature analysis and semi structured expert interviews to answer this research question. In the systematic literature analysis, we identify and analyze a heterogeneous set of 21 AI use cases across five different HC application fields and derive 15 business objectives and six value propositions for HC organizations. We then evaluate and refine the categorized business objectives and value propositions with insights from 11 expert interviews. Our study contributes to research on the value creation mechanism of AI applications in the HC context. Moreover, our results have managerial implications for HC organizations since they can draw on our results to evaluate AI applications, assess investment decisions, and align their AI application portfolio toward an overarching strategy.

In what follows, this study first grounds on relevant work to gain a deeper understanding of the underlying constructs of AI in HC. Next, we describe our qualitative research method by describing the process of data collection and analysis, followed by our derived results on capturing AI applications’ value proposition in HC. Afterward, we discuss our results, including this study’s limitations and pathways for further research. Finally, we summarize our findings and their contribution to theory and practice in the conclusion.

### Relevant work

In the realm of AI, a thorough exploration of its key subdiscipline, machine learning (ML), is essential [24, 25]. ML is a computational model that learns from data without explicitly programming the data [24] and can be further divided into supervised, unsupervised, and reinforcement learning [26]. In supervised learning, the machine undergoes training with labeled data, making it well-suited for tasks involving regression and classification problems [27]. In contrast, unsupervised learning is designed to automatically identify patterns within unlabeled datasets [28], with its primary utility lying in the extraction of features [11]. Reinforcement learning, characterized as a method of systematic experimentation or trial and error, involves a situated agent taking specific actions and observing the rewards it gains from those actions, facilitating the learning of behavior in a given environment [29]. The choice of which type of ML will be used in the different application areas depends on the specific problem, the availability of labeled data, and the nature of the desired outcome.

In recent years, the rapid advances in AI have triggered a revolution in various areas, with numerous impressive advantages. In the financial sector, AI applications can significantly improve security by detecting anomalies and preventing fraud [30]. Within education, AI has emerged as a powerful tool for tailoring learning experiences, aiming to enhance engagement, understanding, and retention [31]. In the energy market, the efficacy of AI extends to fault detection and diagnosis in building energy systems, showcasing its robust capabilities in ensuring system integrity [32]. Moreover, the HC industry is expected to be a promising application area for AI applications. The HC sector is undergoing a significant transformation due to the increasing adoption of digital technologies, with AI technologies at the forefront of this shift. The increasing relevance of AI technologies in HC is underlined by a growing and multidisciplinary stream of AI research, as highlighted by Secinaro et al. [33]. Taking a closer look at the different application areas in HC, AI applications offer promising potential, as demonstrated by the following exemplary AI use cases. In diagnosis, AI applications can identify complex patterns in medical image data more accurately, resulting in precise and objective disease recognition. This can improve patient safety by reducing the risks of misinterpretation [5]. Another use case can be found in biomedical research. For example, AI technology is commonly used for de novo drug design. AI can rapidly browse through molecule libraries to detect nearly ${10}^{60}$ drug-like molecules, accelerating the drug development process [6]. Furthermore, AI applications are used in clinical administration. They enable optimized operation room capacities by automating the process and by including information about absence or waiting times, as well as predicting interruptions [34]. Furthermore, AI applications are used in therapy by predicting personalized medication dosages. As this helps to reduce the mortality risk, it leads to enhanced patient outcomes and quality of care [35]. Intelligent prostheses by which patients can improve interactions are another use case. The AI algorithm continuously detects and classifies myoelectric signal patterns to predict movements, leading to reduced training expenditure and more self-management by the patient [36]. In summary, envisioning that AI applications successfully address persisting challenges, such as lack of transparency (e.g., [37], bias (e.g., [38], privacy concerns, and trust issues (e.g., [39], the potential of AI applications is vast. The conceivable benefits extend to individual practitioners and HC organizations, including hospitals, enabling them to harness AI applications for creating business value and ultimately enhancing competitiveness. Thereby, we follow Schryen’s (p. 141) revisited definition of business value of technologies: “the impact of investments on the multidimensional performance and capabilities of economic entities at various levels, complemented by the ultimate meaning of performance in the economic environment” [40]. His perspective includes all kinds of tangible value (such as an increase in productivity or reduced costs) to intangible value (such as service innovation or customer satisfaction), as well as internal value for the HC organizations and external value for stakeholders, shareholders, and customers. To create business value, it is essential to have a clear understanding of how the potential of AI applications can be captured. The understanding of how information systems, in general, create value is already covered in the literature. For example, Badakhshan et al. [31] focus on how process mining can pave the way to create business value. Leidner et al. [32] examine how enterprise social media adds value for new employees, and Lehrer et al. [33] answer the question of how big data analytics can enable service. There are also studies focusing on the value creation of information systems in the context of HC. For instance, the study by Haddad and Wickramasinghe [41] shows that information technology in HC can capture value by improving the quality of HC delivery, increasing safety, or offering additional services. Strong et al. [42] analyze how electronic health records afford value for HC organizations and determine goal-oriented actions to capture this potential. There is even literature on how machine learning adds value within the discipline of radiology (e.g., [43].

However, these studies either do not address the context of HC, consider technologies other than AI or information systems in general, or focus only on a small area of HC (e.g., radiology) and a subset of AI technology (e.g., machine learning). Although these studies deliver valuable insights into the value creation of information systems, a comprehensive picture of how HC organizations can capture business value with AI applications is missing.

## Method

To answer our research question, we adopted a qualitative inductive research design. This research design is consistent with studies that took a similar perspective on how technologies can create business value [44]. In conducting our structured literature review, we followed the approach of Webster and Watson [45] and included recommendations of Wolfswinkel et al. [46] when considering the inclusion and exclusion criteria. We started by collecting relevant data on different successful AI use cases across five application areas in HC. Siggelkow [47] argued that use cases are able to provide persuasive arguments for causal relationships. In an initial literature screening, we identified five promising application domains focusing on AI applications for patients and HC providers: disease diagnostics (DD) (e.g., [5], biomedical research (BR) (e.g., [6], clinical administration (CA) (e.g., [7], therapy (T) (e.g., [8], and intelligent robotics (IR) (e.g., [9]. Second, to sample AI use cases, we aimed to collect a heterogeneous set of AI use cases within these application domains and consider the heterogeneity in AI applications, underlying data, innovation types, and implementation stages when selecting 21 AI use cases for our in-depth analysis. The AI use case and an exemplary study for each use case are listed in Table 1.

**Table 1:** Analyzed AI use cases

AI use cases	Exemplary study
DD1: Automated image recognition	[5]
DD2: Staging of cancer	[48]
DD3: Objective assessment in image interpretation	[48]
DD4: Genomic cancer therapy	[49]
DD5: Voice analysis for Parkinson’s disease	[50]
DD6: Electroencephalography analysis to detect seizures	[51]
DD7: Facial analysis for detection of rare disease	[52]
BR1: De novo drug design	[6]
BR2: Predictive biomarkers in aging for drug development	[53]
BR3: De-identification of private health information	[54]
BR4: Genomic splicing in research	[55]
CA1: Emergency triage	[56]
CA2: Predictions of mortality in the intensive care unit	[57]
CA3: Operating room scheduling	[34]
CA4: Automated text summarization	[58]
T1: Prediction of the required insulin	[8]
T2: Prediction of vasopressor medication dosage	[35]
T3: Chatbots for patients	[59]
IR1: Intelligent prosthesis	[36]
IR2: AI-based surgery robots	[60]
IR3: Workflow detection for human–robot surgery	[61]

After sampling the AI use cases, we used PubMed to identify papers for each use case. PubMed is recognized as a common database for biomedical and medical research for HC topics in the information systems domain (e.g., [62, 63]. Our search included journal articles, clinical conferences, clinical studies, and comparative studies in English as of 2010. Based on the AI use case sample, we derived a search string based on keywords [45] considering titles and abstracts by following Shepherd et al. [62] guidelines. It was aimed to narrow and specific selection to increase data collection replicability for the use cases. Boolean operators (AND, OR) are used to improve results by combining search terms [62].

((artificial intelligence AND (radiology OR (cancer AND imaging) OR (radiology AND error) OR (cancer AND genomics) OR (speech AND cognitive AND impairment) OR (voice AND parkinson) OR EEG OR (facial AND analysis) OR (drug AND design) OR (Drug AND Biomarker) OR De-identification OR Splicing OR (emergency AND triage) OR (mortality AND prediction) OR (operating AND room) OR text summarization OR (artificial AND pancreas) OR vasopressor OR Chatbot OR (myoelectric prosthesis) OR (automated surgery task) OR (surgery AND workflow)))

**Fig. 1:**
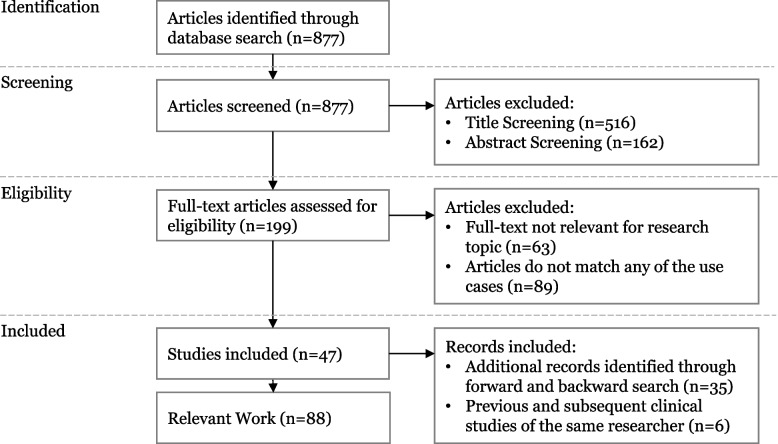
Search strategy

The initial search led to 877 results (see Fig. 1). After title screening, we eliminated 516 papers that are not relevant (i.e., not covering a specific AI application, only including the description of AI algorithm, or not including a managerial perspective and the value created by AI applications). We further excluded 162 papers because their abstract is not concurrent with any specific use case (e.g., because they were literature reviews on overarching topics and did not include a specific AI application). We screened the remaining 199 papers for eligibility through two content-related criteria. First, papers need to cover an AI use case’s whole value proposition creation path, including information on data, algorithms, functions, competitive advantage, and business value of a certain AI application. The papers often only examine how a certain application works but lack the value proposition perspective, which leads to the exclusion of 63 articles. Second, we removed 89 papers that do not match any of our use cases. This step led to a remaining set of 47 relevant papers. During a backward-forward search according to Webster and Watson [45] and Levy and Ellis [64], we additionally included 35 papers. We also incorporated previous and subsequent clinical studies of the same researcher, resulting in an additional six papers. The final set contains 88 relevant papers describing the identified AI use cases, whereby at least three papers describe each AI use case.

In the second step, we engaged in open, axial, and selective coding of the AI use cases following analysis techniques of grounded theory [65]. We focused on extracting business objectives, detailing how each AI application drives value. We documented these for each AI use case by recording codes of business objectives and value propositions and assigning relationships among the open codes. For example, from the following text passage of Berlyand et al. [56], who investigate the use case CA1: “Rapidly interpreting clinical data to classify patients and predict outcomes is paramount to emergency department operations, with direct impacts on cost, efficiency, and quality of care”, we derived the code *rapid task execution.*

After analyzing the AI use cases, we revised the documented tuples to foster consistency and comparability. Then, we iteratively coded the identified tuples by relying on selective coding techniques which is a process to identify and refine categories at a highly generalizable degree [65]. In all 14 coding iterations, one author continuously compares, relates, and associates categories and properties and discusses the coding results with another author. We modified some tuples during the coding process in two ways. First, we equalized small phrasing disparities for homogenous and refined wording. Second, we carefully adjusted the tuples regarding coherency. Finally, we reviewed the coding schema for internal validity through a final comparison with the data [66]. Then, we set the core variables “business objectives” and “value propositions”. We refer to business objectives as improvements through implementing the technology that drives a value proposition. We define value proposition as the inherent commitment to deliver reciprocal value to the organization, its customers, and/or partners [67].

In the third step following Schultze and Avital [68], we conducted semi structured expert interviews to evaluate and refine the value propositions and business objectives. We developed and refined an interview script following the guidelines of Meyers and Newman [69] for qualitative interviews. An additional file shows the used interview script (see Additional file 1). We conducted expert sampling to select suitable interviewees [70]. Due to the interdisciplinarity of the research topic, we chose experts in the two knowledge areas, AI and HC. In the process of expert selection, we ensured that interviewees possessed a minimum of two years of experience in their respective fields. We aimed for a well-balanced mix of diverse professions and positions among the interviewees. Additionally, for those with a primary background in HC, we specifically verified their proficiency and understanding of AI, ensuring a comprehensive perspective across the entire expert panel. Table 2 provides an overview of our expert sample. The interviewees were recruited in the authors’ networks and by cold calling. Identified experts were first contacted by email, including some brief information regarding the study. If there was no response within two weeks, they were contacted again by telephone to arrange an interview date. In total, we conducted 11 interviews that took place in a time range between 40 and 75 min. The expert interviews are transcribed verbatim using the software f4. As a coding aid, we use the software MAXQDA—a tool for qualitative data analysis which is frequently used in the analyses of qualitative data in the HC domain (e.g., [38, 71, 72]).

**Table 2:** Overview of interviewees

Expert	Expertise	Position	Experience
E1	HC	Consultant & Professor in innovations in HC	8 years
E2	AI and HC	Senior Analyst HC investments	5 years
E3	AI and HC	Co-Founder of an AI-based conversational agent startup in HC section	2 years
E4	AI and HC	Director of AI strategy in a HC company	6 years
E5	AI and HC	Innovation Manager of HC startups	4 years
E6	AI and HC	Product Manager of AI-based image diagnostic solutions	5 years
E7	AI and HC	Co-Founder & Software Engineer of an AI-based clinical administration startup	3 years
E8	AI and HC	Senior Physician of Anesthesia	17 years
E9	AI and HC	Data Scientist of clinical administration company	6 years
E10	HC	Assistant Manager in digital HC innovations	4 years
E11	AI and HC	Head of Entrepreneurship & Tech Education	11 years

## Results

**Fig. 2:**
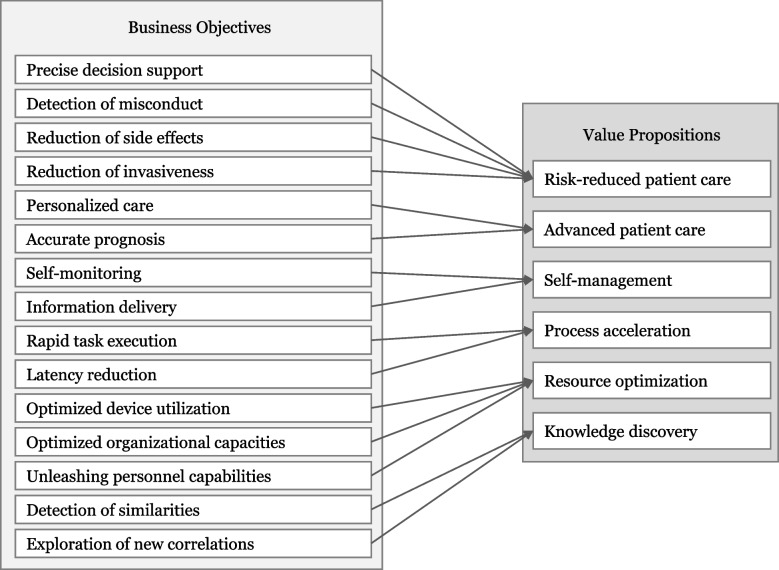
Business objectives and value propositions risk-reduced patient care

To systematically decompose how HC organizations can realize value propositions from AI applications, we identified 15 business objectives and six value propositions (see Fig. 2). These business objectives and value propositions resulted from analyzing the collected data, which we derived from the literature and refined through expert interviews. In the following, we describe the six value propositions and elaborate on how the specific AI business objectives can result in value propositions. This will be followed by a discussion of the results in the discussion of the paper.

This value proposition follows business objectives that may identify and reduce threats and adverse factors during medical procedures. HC belongs to a high-risk domain since there are uncertain external factors (E4), including physicians’ fatigue, distractions, or cognitive biases [73, 74]. AI applications can reduce certain risks by enabling precise decision support, detecting misconduct, reducing emergent side effects, and reducing invasiveness.

Precise decision support stems from AI applications’ capability to integrate various data types into the decision-making process, gaining a sophisticated overview of a phenomenon. Precise knowledge about all uncertainty factors reduces the ambiguity of decision-making processes [49]. E5 confirms that AI applications can be seen as a “perceptual enhancement”, enabling more comprehensive and context-based decision support. Humans are naturally prone to innate and socially adapted biases that also affect HC professionals [14]. Use Case CA1 highlights how rapid decision-making by HC professionals during emergency triage may lead to overlooking subtle yet crucial signs. AI applications can offer decision support based on historical data, enhancing objectivity and accuracy [56].

Detection of misconduct is possible since AI applications can map and monitor clinical workflows and recognize irregularities early. In this context, E10 highlights that “one of the best examples is the interception of abnormalities.” For instance, AI applications can assist in allocating medications in hospitals (Use case T2). Since HC professionals can be tired or distracted in medication preparation, AI applications may avoid serious consequences for patients by monitoring allocation processes and patients’ reactions. Thus, AI applications can reduce abuse and increase safety.

Reduction of emergent side effects is enabled by AI applications that continuously monitor and process data. If different treatments and medications are combined during a patient’s clinical pathway, it may cause overdosage or evoke co-effects and comorbidities, causing danger for the patient [75]. AI applications can prevent these by detecting and predicting these effects. For instance, AI applications can calculate the medication dosage for the individual and predict contraindications (Use case T2) [76]. E3 adds that the reduction of side effects also includes “cross-impacts between medications or possible symptoms that only occur for patients of a certain age or disease.” Avoidable side effects can thus be detected at an early stage, resulting in better outcomes.

Reduction of invasiveness of medical treatments or surgeries is possible by allowing AI applications to compensate for and overcome human weaknesses and limitations. During surgery, AI applications can continuously monitor a robot’s position and accurately predict its trajectories [77]. Intelligent robots can eliminate human tremors and access hard-to-reach body parts [60]. E2 validates, “a robot does not tremble; a robot moves in a perfectly straight line.” The precise AI-controlled movement of surgical robots minimizes the risk of injuring nearby vessels and organs [61]. Use cases DD5 and DD7 elucidate how AI applications enable new methods to perform noninvasive diagnoses. Reducing invasiveness has a major impact on the patient’s recovery, safety, and outcome quality.

### Advanced patient care

Advanced patient care follows business objectives that extend patient care to increase the quality of care. One of HC’s primary goals is to provide the most effective treatment outcome. AI applications can advance patient care as they enable personalized care and accurate prognosis.

Personalized care can be enabled by the ability of AI technologies to integrate and process individual structured and unstructured patient data to increase the compatibility of patient and health interventions. For instance, by analyzing genome mutations, AI applications precisely assess cancer, enabling personalized therapy and increasing the likelihood of enhancing outcome quality (Use case DD4). E11 sums up that “we can improve treatment or even make it more specific for the patient. This is, of course, the dream of healthcare”. Use case T1 exemplifies how the integration of AI applications facilitates personalized products, such as an artificial pancreas. The pancreas predicts glucose levels in real time and adapts insulin supplementation. Personalized care allows good care to be made even better by tailoring care to the individual.

Accurate prognosis is achieved by AI applications that track, combine, and analyze HC data and historical data to make accurate predictions. For instance, AI applications can precisely analyze tumor tissue to improve the stratification of cancer patients. Based on this result, the selection of adjuvant therapy can be refined, improving the effectiveness of care [48]. Use case DD6 shows how AI applications can predict seizure onset zones to enhance the prognosis of epileptic seizures. In this context, E10 adds that an accurate prognosis fosters early and preventive care.

### Self-management

Self-management follows the business objectives that increase disease controllability through the support of intelligent medical products. AI applications can foster self-management by self-monitoring and providing a new way of delivering information.

Self-monitoring is enhanced by AI applications, which can automatically process frequently measured data. There are AI-based chatbots, mobile applications, wearables, and other medical products that gather periodic data and are used by people to monitor themselves in the health context (e.g., [78, 79]. Frequent data collection of these products (e.g., using sensors) enables AI applications to analyze periodic data and become aware of abnormalities. While the amount of data rises, the applications can improve their performance continuously (E2). Through continuous tracking of heartbeats via wearables, AI applications can precisely detect irregularities, notify their users in the case of irregularities, empower quicker treatment (E2), and may reduce hospital visits (E9). Self-monitoring enhances patient safety and allows the patient to be more physician-independent and involved in their HC.

Information delivery to the patient is enabled by AI applications that give medical advice adjusted to the patient’s needs. Often, patients lack profound knowledge about their anomalies. AI applications can contextualize patients’ symptoms to provide anamnesis support and deliver interactive advice [59]. While HC professionals must focus on one diagnostic pathway, AI applications can process information to investigate different diagnostic branches simultaneously (E5). Thus, these applications can deliver high-quality information based on the patient’s feedback, for instance, when using an intelligent conversational agent (use case T3). E4 highlights that this can improve doctoral consultations because “the patient is already informed and already has information when he comes to talk to doctors”.

### Process acceleration

Process acceleration comprises business objectives that enable speed and low latencies. Speed describes how fast one can perform a task, while latency specifies how much time elapses from an event until a task is executed. AI applications can accelerate processes by rapid task execution and reducing latency.

Rapid task execution can be achieved by the ability of AI applications to process large amounts of data and identify patterns in a short time. In this context, E4 mentions that AI applications can drill diagnosis down to seconds. For instance, whereas doctors need several minutes for profound image-based detection, AI applications have a much faster report turnaround time (use case DD1). Besides, rapid data processing also opens up new opportunities in drug development. AI applications can rapidly browse through molecule libraries to detect nearly 10^60 molecules, which are synthetically available (use case BR1). This immense speed during a discovery process has an essential influence on the business potential and can enormously decrease research costs (E10).

Latency reduction can be enabled by AI technologies monitoring and dynamically processing information and environmental factors. By continuously evaluating vital signs and electrocardiogram records, AI applications can predict the in-house mortality of patients in real time [57]. The AI application can detect an increased mortality risk faster than HC professionals, enabling a more rapid emergency intervention. In this case, AI applications decrease the time delay between the cause and the reaction, which positively impacts patient care. E7 emphasizes the importance of short latencies: “One of the most important things is that the timeframe between the point when all the data is available, and a decision has been made, […] must be kept short.”

### Resource optimization

Resource optimization follows the business objectives that manage limited resources and capacities. The HC industry faces a lack of sufficient resources, especially through a shortage of specialists (E8), which in turn negatively influences waiting times. AI applications can support efficient resource allocation by optimizing device utilization, organizational capacities and unleashing personnel capabilities.

Optimized device utilization can be enhanced by AI applications that track, analyze, and precisely predict load of times of medical equipment in real-time. For instance, AI applications can maximize X-Ray or magnetic resonance tomography device utilization (use case CA3). Besides, AI applications can enable a dynamic replanning of device utilization by including absence or waiting times and predicting interruptions. Intelligent resource optimization may include various key variables (e.g., the maximized lifespan of a radiation scanner) [48]. Optimized device utilization reduces the time periods when the device is not utilized, and thus, losses are made.

Optimized organizational capacities are possible due to AI applications breaking up static key performance indicators and finding more dynamic measuring approaches for the required workflow changes (E5, E10). The utilization of capacities in hospitals relies on various known and unknown parameters, which are often interdependent [80]. AI applications can detect and optimize these dependencies to manage capacity. An example is the optimization of clinical occupancy in the hospital (use case CA3), which has a strong impact on cost. E5 adds that the integration of AI applications may increase the reliability of planning HC resources since they can predict capacity trends from historical occupancy rates. Optimized planning of capacities can prevent capacities from remaining unused and fixed costs from being offset by no revenue.

Unleashing personnel capabilities is enabled by AI applications performing analytical and administrative tasks, relieving caregivers’ workload (E8, E10, E11). E7 validates that “our conviction is […] that administrational tasks generate the greatest added value and benefit for doctors and caregivers.” Administrative tasks include the creation of case summaries (use case CA4) or automated de-identification of private health information in electronic health records (use case BR2) [54]. E8 says that resource optimization enables “more time for direct contact with patients.”

### Knowledge discovery

Knowledge discovery follows the business objectives that increase perception and access to novel and previously unrevealed information. AI applications might synthesize and contextualize medical knowledge to create uniform or equalized semantics of information (E5, E11). This semantics enables a translation of knowledge for specific users.

Detection of similarities is enabled by AI applications identifying entities with similar features. AI applications can screen complex and nonlinear databases to identify reoccurring patterns without any a priori understanding of the data (E3). These similarities generate valuable knowledge, which can be applied to enhance scientific research processes such as drug development (use case BR1). In drug development, AI applications can facilitate ligand-based screening to detect new active molecules based on similarities compared with already existing molecular properties. This increases the effectiveness of drug design and reduces risks in clinical trials [6].

Exploration of new correlations is facilitated by AI applications identifying relationships in data. In diagnostics, AI applications can analyze facial photographs to accurately identify genotype–phenotype correlations and, thus, increase the detection rate of rare diseases (use case DD7). E8 states the potential of AI applications in the field of knowledge discovery: “Well, if you are researching in any medical area, then everybody aims to understand and describe phenomena because science always demands a certain causation.” However, it is crucial to develop transparent and intelligible inferences that are comprehensible for HC professionals and researchers. Exploring new correlations improves diagnoses of rare diseases and ensures earlier treatment.

After describing each business objective and value proposition, we summarize the AI use cases’ contributions to the value propositions in Table 3.

**Table 3:** Value propositions of AI use cases

AI use cases	Value proposition					
	**Risk-reduced patient care**	**Advanced** **patient care**	**Self-management**	**Process acceleration**	**Resource optimization**	**Knowledge discovery**
DD1: Automated image recognition	x			x	x	
DD2: Staging of cancer	x	x				
DD3: Objective assessment in image interpretation	x			x		
DD4: Genomic cancer therapy		x				
DD5: Voice analysis for Parkinson’s disease	x	x	x			
DD6: Electroencephalography analysis to detect seizures	x	x			x	
DD7: Facial analysis for detection of rare disease		x		x		x
BR1: De novo drug design	x			x		x
BR2: Predictive biomarkers in aging for drug development	x	x				x
BR3: De-identification of private health information				x	x	
BR4: Genomic splicing in research		x		x		x
CA1: Emergency triage	x			x	x	
CA2: Predictions of mortality in the intensive care unit	x	x		x		
CA3: Operating room scheduling					x	
CA4: Automated text summarization				x	x	
T1: Prediction of the required insulin	x	x	x			
T2: Prediction of vasopressor medication dosage	x	x				
T3: Chatbots for patients		x	x		x	
IR1: Intelligent prosthesis		x	x			
IR2: AI-based surgery robots	x	x		x		
IR3: Workflow detection for human–robot surgery	x			x		
**Number of entries**	**13**	**13**	**4**	**11**	**6**	**4**

## Discussion

By revealing 15 business objectives that translate into six value propositions, we contribute to the academic discourse on the value creation of AI (e.g. [81] and provide prescriptive knowledge on AI applications' value propositions in the HC domain. Our discourse also emphasizes that our findings are not only relevant to the field of value creation research but can also be helpful for adoption research. The value propositions we have identified can be a good starting point to accelerate the adoption of AI in HC, as the understanding of potential value propositions that we foster could mitigate some of the current obstacles to the adoption of AI applications in HC. For example, our findings may help to mitigate the obstacle “added value”, which is presented in the study by Hennrich et al.38 [38] as users’ concerns that AI might create more burden than benefits.

Further, we deliver valuable implications for practice and provide a comprehensive picture of how organizations in the context of HC can achieve business value with AI applications from a managerial level, which has been missing until now. We guide HC organizations in evaluating their AI applications or those of the competition to assess AI investment decisions and align their AI application portfolio toward an overarching strategy. These results will foster the adoption of AI applications as HC organizations can now understand how they can unfold AI applications’ capabilities into business value. In case a hospital’s major strategy is to reduce patient risks due to limited personal capacities, it might be beneficial for them to invest in AI applications that reduce side effects by calculating medication dosages (use case T2). If an HC organization currently faces issues with overcrowded emergency rooms, the HC organization might acquire AI applications that increase information delivery and help patients decide if and when they should visit the hospital (use case T3) to increase patients’ self-management and, in turn, improve triage. Besides, our findings also offer valuable insights for AI developers. Addressing issues such as transparency and the alignment of AI applications with the needs of HC professionals is crucial. Adapting AI solutions to the specific requirements of the HC sector ensures responsible integration and thus the realization of the expected values.

A closer look at the current challenges in the HC sector reveals that new solutions to mitigate them and improve value creation are needed. Given that a nurse, for example, dedicates a substantial 25% of their working hours to administrative tasks [17], the rationale behind the respondents’ (E7) recognition of “the greatest added value” in utilizing AI applications for administrative purposes becomes evident. The potential of AI applications in streamlining administrative tasks lies in creating additional time for meaningful patient interactions. Acknowledging the significant impact of the doctor-patient interpersonal relationship on both the patient’s well-being and the processes of diagnosis and healing, as elucidated by Buck et al. [82] in their interview study, the physicians interviewed emphasized that the mere presence of the doctor in the same room often alleviates the patient’s problems. Consequently, it becomes apparent that the intangible value of AI applications plays a crucial role in the context of HC and is an important factor in the investment decision as to where an AI application should be deployed.

The interviews also indicate that the special context of the HC sector leads to concerns regarding the use of AI applications. For example, one interviewee emphasized a fundamental characteristic of medical staff by pointing out that physicians have a natural desire to understand all phenomena (E8). AI applications, however, are currently struggling with the challenge of transparency. This challenge is described by the so-called black box problem, a phenomenon that makes it impossible to decipher the underlying algorithms that lead to a particular recommendation [37]. The lack of transparency and the resulting lack of intervention options for medical staff can lead to incorrect decisions by the AI application, which may cause considerable damage. Aware of these risks, physicians are currently struggling with trust issues in AI applications [72]. The numerous opportunities for value creation through AI applications in HC are offset by the significant risk of causing considerable harm to patients if the technology is not yet fully mature. Ultimately, it remains essential to keep in mind that there are many ethical questions to be answered [83], and AI applications are still facing many obstacles [38] that must be overcome in order to realize the expected values and avoid serious harm. One important first step in mitigating the obstacles is disseminating the concerns and risks to relevant stakeholders, emphasizing the urgency for collaborative scientific and public monitoring efforts [84]. However, keeping these obstacles in mind, by providing prescriptive knowledge, we enhance the understanding of AI’s value creation paths in the HC industry and thus help to drive AI integration forward. For example, looking at the value proposition *risk reduced patient care*, we demonstrate that this value proposition is determined by four business objectives: *precise decision support*, *detection of misconduct, reduction of side effects,* and *reduction of invasiveness*. Similarly, the AI application’s capability to analyze data more accurately in diagnosis (use case DD1) enables the business objective *precise decision support*, thereby reducing risks in patient care. Another mechanism can be seen, for example, considering the business objective *task execution*, which leads to the value proposition *process acceleration*. The ability of AI applications to rapidly analyze large amounts of data and recognize patterns in biomedical research (use case BR1) allows a faster drug development process.

### Further research

By investigating the value creation mechanism of AI applications for HC organizations, we not only make an important contribution to research and practice but also create a valuable foundation for future studies. While we have systematically identified the relations between the business objectives and value propositions, further research is needed to investigate how the business objectives themselves are determined. While the examination of AI capabilities was not the primary research focus, we found first evidence in the use cases that indicates AI technology’s unique capabilities (e.g., to make diagnoses accurate, faster, and more objective) that foster one or several business objectives (e.g., rapid task execution, precise decision support) and unlock one or several value propositions (e.g., *Risk-reduced patient care, process acceleration*). In subsequent research, we aim to integrate these into the value creation mechanism by identifying which specific AI capabilities drive business objectives, thereby advancing the understanding of how AI applications in HC create value propositions.

### Limitations

This study is subject to certain limitations of methodological and conceptual nature. First, while our methodological approach covers an in-depth analysis of 21 AI use cases, extending the sample of AI use cases would foster the generalizability of the results. This is especially important regarding the latest developments on generative AI and its newcoming use cases. However, our results demonstrate that these AI use cases already provide rich information to derive 15 business objectives, which translate into six value propositions. Second, while many papers assume the potential of AI applications to create value propositions, only a few papers explicitly focus on the value creation and capture mechanisms. To compensate for this paucity of appropriate papers, we used 11 expert interviews to enrich and evaluate the results. Besides, these interviews ensured the practical relevance and reliability of the derived results. Third, we acknowledge limitations of conceptual nature. Our study predominantly takes an optimistic perspective on AI applications in medicine. While we discuss the potential benefits and value propositions in detail, it is important to emphasize that there are still significant barriers and risks currently associated with AI applications that need to be addressed before the identified values can be realized. Furthermore, our investigation is limited because we derive the expected value of AI applications without having extensive real-world use cases to evaluate. It is important to emphasize that our findings are preliminary, and critical reassessment will be essential as the broader implementation of AI applications in medical practice progresses. These limitations emphasize the need for ongoing research and monitoring to understand the true value of AI applications in HC fully.

## Conclusions

This study aimed to investigate how AI applications can create value for HC organizations. After elaborating on a diverse and comprehensive set of AI use cases, we are confident that AI applications can create value by making HC, among others, more precise, individualized, self-determined, faster, resource-optimized, and data insight-driven. Especially with regard to the mounting challenges of the industry, such as the aging population and the resulting increase in HC professionals’ workloads, the integration of AI applications and the expected benefits have become more critical than ever. Based on the systematic literature review and expert interviews, we derived 15 business objectives that translate into the following six value propositions that describe how HC organizations can capture the value of AI applications: *risk-reduced patient care, advanced patient care, self-management, process acceleration, resource optimization,* and *knowledge discovery*.

By presenting and discussing our results, we enhance the understanding of how HC organizations can unlock AI applications’ value proposition. We provide HC organizations with valuable insights to help them strategically assess their AI applications as well as those deployed by competitors at a management level. Our goal is to facilitate informed decision-making regarding AI investments and enable HC organizations to align their AI application portfolios with a comprehensive and overarching strategy. However, even if various value proposition-creating scenarios exist, AI applications are not yet fully mature in every area or ready for widespread use. Ultimately, it remains essential to take a critical look at which AI applications can be used for which task at which point in time to achieve the promised value. Nonetheless, we are confident that we can shed more light on the value proposition-capturing mechanism and, therefore, support AI application adoption in HC.

### Supplementary Information

**Supplementary Material 1. **

## Data Availability

The datasets analyzed during the current study are available from the corresponding author on reasonable request.

## Abbreviations

AI: Artificial Intelligence
HC: Healthcare
ML: Machine Learning
