# Supplementary material for: Capturing artificial intelligence applications’ value proposition in healthcare – a qualitative research study
Source: BMC Health Serv Res. 2024 Apr 3;24:420. doi: 10.1186/s12913-024-10894-4 (PMC10993548; doi:10.1186/s12913-024-10894-4)
Supplement: Supplementary file 1 — Supplementary Material 1. [file 12913_2024_10894_MOESM1_ESM.pdf]

Additional file 1

Interview script

Date:

Name of Interviewee:

Industry:

Position:

1. **Introduction**

    1.1.Administrative introduction

    1.2.Instructions for the interview process

    1.3.Introduction to the research project

2. **Characterization of the expert**

    2.1.Professional experience: How many years of professional experience do you have?

    2.2.AI - Expertise: Because there exist many different understandings. Just briefly, what do you understand by AI?

    If necessary, definition of AI and ML

    Artificial intelligence are intelligent agents that can solve problems independently and act on them, i.e., they can learn by trial and error. Currently the most common machine learning applications (sub-category of AI). These are methods that use learning to recognize connections in existing data sets to make predictions based on them.

3. **Evaluation of AI's Action Potentials in the Health Care domain**

Explanation of the application domains in health care

Definition and example of possible action potentials

By action possibilities, I understand the benefit that artificial intelligence brings in the health care domain. What can AI actually do? How can AI be applied?

Example: AI in industrial production – processes can be automated and are faster

3.1.*Requesting the action possibilities of AI*

3.1.1. Which action possibilities do you see for the use of AI in the health care domain?

Introduction to affordance classes

3.2. Understandability

3.2.1. Are the action potentials understandable according to your expertise?

3.3. Completeness

3.3.1. Could you add further action potentials of AI?

3.3.2. So then, are the action potentials complete from your view?

3.3.3. Are there any action potentials with which you do not agree?

3.4. Relevance

3.4.1. Which three affordances are particularly relevant in the health care sector? Why?

4. Evaluation of Business Levers in Healthcare

Introduction to the Business Levers

4.1. Understandability

4.1.1. Are the business levers understandable from your perspective?

4.2. Completeness

4.2.1. Could you add additional business levers?

4.2.2. Are the business levers complete according to your perspective?

4.2.3. Are there any business levers that you disagree with?

4.3. Relevance

4.3.1. Which three business levers are the most relevant for innovations in the healthcare sector? Why?

5. Evaluation of the effect path model

5.1 Level of detail and comprehensibility

5.1.1 Is the model understandable?

5.1.2. Is the model neither too detailed nor too undetailed?

5.2. Internal consistency (Is the model consistent in itself?)

5.2.1. Are all constructs and their relationships clear and unambiguous?

5.2.2. Does the model contradict itself?

5.3. Relevance

5.3.1 Does the model address a relevant problem?

5.4 Consistency with real world phenomena and completeness

5.4.1. Disclosure of the value creation of real use cases: Is the model able to show the emergence of business potential (value) through AI applications in practice?

5.4.2 Completeness: Is there anything missing in the model to show the creation of business potential (value) through AI applications?

5.4.3 Is the model applicable in actual practice?

5.5. Robustness (the model remains valid across AI applications and domains)

5.5.1 Universal applicability: Do you see reasons why the model should not be applicable beyond different AI applications and domains? For example, do you see a reason why it should not be applicable in other industries?

Concluding statement
